# gEMpicker: a highly parallel GPU-accelerated particle picking tool for cryo-electron microscopy

**DOI:** 10.1186/1472-6807-13-25

**Published:** 2013-10-21

**Authors:** Thai V Hoang, Xavier Cavin, Patrick Schultz, David W Ritchie

**Affiliations:** 1 Inria Nancy - Grand Est, 615 rue du Jardin Botanique, 54600 Villers-lès-Nancy, France; 2 IGBMC, 1 rue Laurent Fries, 67404 Illkirch, France

**Keywords:** Cryo-EM particle picking, Graphics processor units, Normalised cross-correlation, Fast Fourier transform, Parallel computing, Tree-based reduction

## Abstract

**Background:**

Picking images of particles in cryo-electron micrographs is an important step in solving the 3D structures of large macromolecular assemblies. However, in order to achieve sub-nanometre resolution it is often necessary to capture and process many thousands or even several millions of 2D particle images. Thus, a computational bottleneck in reaching high resolution is the accurate and automatic picking of particles from raw cryo-electron micrographs.

**Results:**

We have developed “gEMpicker”, a highly parallel correlation-based particle picking tool. To our knowledge, gEMpicker is the first particle picking program to use multiple graphics processor units (GPUs) to accelerate the calculation. When tested on the publicly available keyhole limpet hemocyanin dataset, we find that gEMpicker gives similar results to the FindEM program. However, compared to calculating correlations on one core of a contemporary central processor unit (CPU), running gEMpicker on a modern GPU gives a speed-up of about 27 ×. To achieve even higher processing speeds, the basic correlation calculations are accelerated considerably by using a hierarchy of parallel programming techniques to distribute the calculation over multiple GPUs and CPU cores attached to multiple nodes of a computer cluster. By using a theoretically optimal reduction algorithm to collect and combine the cluster calculation results, the speed of the overall calculation scales almost linearly with the number of cluster nodes available.

**Conclusions:**

The very high picking throughput that is now possible using GPU-powered workstations or computer clusters will help experimentalists to achieve higher resolution 3D reconstructions more rapidly than before.

## Background

Despite recent advances in the use of computational techniques, solving the structures of large macromolecular complexes by cryo-electron microscopy (EM) is still a painstaking and labour-intensive task [1]. It is also a very computationally intensive task. In single-particle cryo-EM, large numbers of micrographs containing low-resolution and noisy two-dimensional (2D) images of the particle of interest are recorded. Because each micrograph usually contains multiple particles in multiple random orientations, and possibly also in various conformations, the particles are then picked and classified into groups having similar orientations. Fast Fourier transform (FFT) deconvolution and averaging techniques may then be applied to reduce both systematic deformations of the 2D images due to the instrument’s contrast transfer function and the random noise which arises from using low electron intensities necessary to preserve the structural integrity of the samples. Once a good set of 2D images has been obtained, a three-dimensional (3D) electron density map of the particle may be constructed using 3D back-projection or Radon transform techniques [2], for example. However, the resolution of such maps, which are often calculated from only *O*(10^4^) molecular images, is low compared to density maps obtained by X-ray crystallography which are typically derived from *O*(10^15^) molecules. Therefore, in cryo-EM, the main way to increase the resolution of the final density map is to capture and process many thousands or even several millions of 2D particle images. In the past, the particles in EM micrographs were picked manually, but this is not practical to reach sub-nanometre resolution or to resolve conformational changes within molecules. Modern digitial imaging technology combined with automated high-throughput data collection techniques now allow both higher resolution and unlimited sizes of 2D datasets to be captured. Hence, a major bottleneck in reaching atomic resolution in 3D reconstruction by cryo-EM is now the accurate and automated picking of particles from the raw EM micrographs.

Many methods have been proposed for automatic cryo-EM particle picking [3,4]. Amongst the most popular are those that use particle templates to facilitate particle recognition. A template is usually a noise-free representation of the particle in a particular orientation. It can be obtained either by projecting a known 3D structure onto a 2D plane or by calculating the average of some representative particles selected from micrographs. Some picking methods use mathematical functions for templates such as the difference of Gaussians method [5,6]. In general, template-based methods recognise particles by computing similarity scores between the template and similar sized regions of each micrograph. For example, a widely used template-based method employs the normalised cross-correlation technique [7] which calculates an array of matching scores in the form of a 2D correlation map. This approach has been implemented in FindEM [8], SPIDER LFCPick [9], and SIGNATURE [10], for example.

Some picking methods use machine learning techniques to discriminate between real particles and non-particles such as those due to contaminants and noise. Example techniques are cascades of classifiers [11,12], pyramid of neural networks [13], and support vector machine [14]. Other methods are based on the observation that 2D images of particles often have rather limited geometric complexity. For example, [15] use a Hough transform for particle edge detection. A related approach uses image processing techniques to segment particles directly from micrographs [16]. However, methods which do not use templates often require human intervention during the picking process.

Because it is difficult to surpass the accuracy of automatic template-based methods when the templates match the particles well, template-based approaches are often preferred although their computational cost is often higher than that of other methods [4]. However, in single particle cryo-EM, large and diverse sets of both micrographs and templates are usually needed to represent and identify different orientations of particles in micrographs in order to achieve a high resolution 3D reconstruction. There is therefore a need to be able to pick multiple images from multiple micrographs using multiple templates as rapidly as possible. In order to help satisfy this need, we have developed a highly parallel correlation-based particle picking tool called gEMpicker, which exploits recent advances in high performance computing technology in order to distribute particle picking calculations over multiple nodes of a computer cluster.

Nowadays, most research institutions have at least one computer cluster for scientific calculations. Each node of the cluster usually consists of several CPU cores, and an increasing number of clusters are configured with a certain number of GPUs in order to accelerate arithmetically intense calculations. Indeed, in the last few years, GPUs have been used to accelerate many scientific calculations [17] in fields ranging from molecular dynamics simulations [18] and quantum chemistry [19] to protein and DNA sequence alignment [20] and protein docking [21]. Recently, GPUs have also been used to accelerate single particle reconstruction [22], tomographic reconstruction [23], and subtomogram averaging [24]. With these observations in mind, we designed gEMpicker to be able to adapt easily to different hardware configurations, ranging from a modest workstation with one or two attached GPUs to large CPU-based or GPU-based clusters with tens or even hundreds of processors, and we have endeavoured to ensure that its performance increases linearly with the computational resources available. Here, we present particle picking speed-up results obtained on four different computational platforms, and we demonstrate the practical utility of the approach using the publicly available keyhole limpet hemocyanin (KLH) dataset. To our knowledge, gEMpicker is the first particle picking program to use multiple modern graphics processor units (GPUs) to accelerate FFT-based NCC calculations.

## Implementation

### NCC-based automatic particle picking

Given a set of search images, *S*_*k*_ (*k*=1,2,…,*N*), each of which contains a candidate particle to be picked, NCC-based automatic particle picking involves determining the highest peaks in the correlation maps calculated between these search images and the target image. The overall calculation involves essentially three main steps. The first step calculates the correlation, NCC_*k*_, between each *S*_*k*_ and the target image. NCC_*k*_ can be efficiently calculated using FFTs by exploiting the formulation in [7] (Additional file 1 Section 1). The second step combines all of the NCC_*k*_ correlation maps into a global correlation map NCC using

(1) $$\text{NCC}(v)\;=\;\underset{k}{\text{max}}\;{\text{NCC}}_{k}(v)$$

for all relative distance **v** of search images to the origin of target image. In the parallel processing community, the process of gathering results in this way is often called a “reduction” because it reduces multiple result arrays into a single global result array. In large-scale distributed calculations, the efficiency of the reduction step can have a significant impact on the overall speed of the calculation. We return to this point below.

In addition to the global correlation map, it is also necessary to store the identity of the search image that gives rise to each local maximum. Hence, a global index map, IND, is calculated along with the global NCC according to

(2) $$\text{IND}(v)\;=\;\underset{k}{\text{argmax}}\;{\text{NCC}}_{k}(v).$$

Assuming that **v** is the location of a local maximum in NCC, the search image that corresponds to that local maximum is given by *k*=IND(**v**). In other words, the calculation has associated the search image *S*_*k*=IND(**v**)_ at location **v** of the target image. Lastly, the third step locates the coordinates of local maxima in NCC in order to produce a final list of picked particles. The above procedure is then repeated for each target image in the dataset.

### FFT size and zero-padding

Because almost all of the computational cost in gEMpicker arises from FFT-based NCC calculations, the choice of FFT library can significantly affect overall performance. We therefore tested gEMpicker using the proprietary MKL (Math Kernel Library) [25], CUFFT (CUDA Fast Fourier Transform) [26], and the open source FFTW (Fastest Fourier Transform in the West) [27] libraries. Although the theoretical advantage of the FFT is that it can perform a calculation that apparently requires *O*(*N*^2^) operations in just *O*(*N* log*N*) time, the actual speed-up that might be achieved can be quite sensitive to the dimension *N*.

Current FFT libraries use the Cooley–Tukey algorithm [28] to reduce recursively a transform of size *N* into transforms of smaller dimensions which are normally implemented as small “kernels” of dimension 2, 3, 5, or 7. If the dimension cannot be factored into small prime numbers, a slower general purpose algorithm is used (e.g. [29,30]). Therefore, if the image dimension is not a natural product of small primes, it is often worthwhile to pad the image with zeros up to a suitable larger dimension. Additionally, on current GPUs, global GPU memory can be accessed most efficiently if memory request can be factored into similar dimensions, because this can allow the GPU to coalesce multiple memory accesses into a single transaction (the precise conditions necessary for coalesced memory access are described in the CUDA C Programming Guide [31]). Consequently, gEMpicker automatically zero-pads images when it detects an opportunity to improve performance due to the above considerations. This simple trick has demonstrated its effectiveness when the data size does not conform to the library’s recommendation.

### Parallel processing framework

In parallel processing, it is usual to use the notion of a “thread” to mean one instance of a calculation that will run essentially independently on one CPU core. Often, multiple threads are launched from a single parent program, or “process”, on each CPU node. Although different threads may run independently, they often still communicate with each other in a controlled way using one or more message passing techniques to send and receive data and results. Here, we consider the basic unit of calculation to be the correlation of one template with one micrograph because this operation is relatively expensive yet it does not depend on either the number of micrographs or the number of templates to be processed. With this level of granularity, the particle picking problem can be parallelised quite naturally by distributing the correlation calculations over several threads running in parallel. When GPUs are available, it is legitimate for a CPU thread to pass a part or even all of a calculation to an attached GPU.

When running in multi-threaded mode, each thread will calculate the correlation between the micrograph and multiple templates. However, concurrent reading of data by multiple threads could cause contention in the disc storage device and consequently lead to sub-optimal performance. Therefore, to avoid this problem, gEMpicker adopts a producer–consumer pattern [32]. The producer’s job is simply to read data from disc, and copy it into a queue. If the number of producers is one, which is the case in gEMpicker, there is only one stream of data from the storage device, and hence the possibility of contention is completely avoided. gEMpicker normally uses multiple consumer threads according to a simple thread pool pattern [33]. Each consumer removes one template at a time from the queue and processes it independently of any other template calculations. In order to avoid race or deadlock conditions amongst the threads, access to the queue is controlled by locks within the “Boost.Thread” library [34]. Additionally, if the queue becomes empty, any idle consumer threads will sleep until more data is made available by the producer. On the other hand, if the queue grows beyond a certain size, the producer will sleep in order to avoid exhausting physical memory. The number of consumer threads in the pool can be adapted according to the available resources. Typically, the number of threads would be set to the number of CPU cores or the number of GPUs per node. Thus, the producer-consumer model provides a way to read data smoothly from disc and to process it as quickly as possible.

In order to calculate the global NCC map for a micrograph with a set of templates, gEMpicker distributes the calculation over a given number of threads, which might ultimately be executed on multiple CPUs, GPUs, or a mixture of the two. Thus each thread *t* calculates NCC_*t*_ for a subset of the templates and it maintains NCC^*t*^ and IND^*t*^ as its individual correlation map and the corresponding index map. When the queue of templates becomes exhausted, each thread combines its NCC^*t*^ with NCC^*p*^ so that NCC^*p*^ and IND^*p*^ will contain the candidate picks calculated by the threads belonging to process *p*. When running on a single workstation, NCC^*p*^ and IND^*p*^ will immediately describe all of the picked particles, and all that remains is to identify the local maxima to obtain the final picked list.

### Cluster implementation

**Figure 1:**
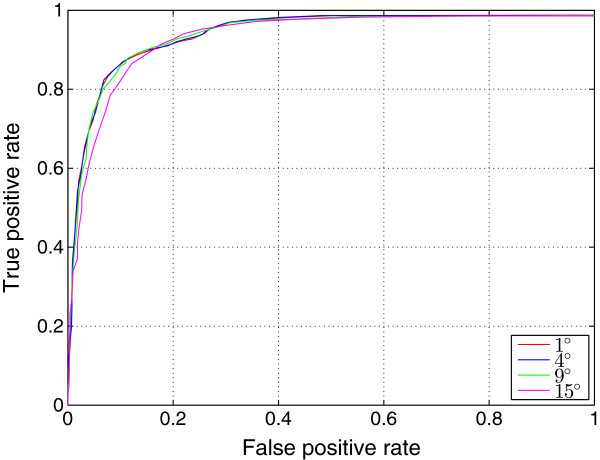
**The hierarchical parallel structure of gEMpicker.** At the top level of the hierarchy, the MPI parallel processing library is used to distribute the calculation over multiple processes on the nodes of a CPU or GPU cluster. On each node, the Boost.Thread library is used to synchronise multiple coarse-grained CPU threads which cooperate using the producer-consumer programming model. During the reduction step, fine-grained parallelisation using OpenMP is used to combine the process-level correlation maps from the CPU nodes, and the MPI_Reduce function is used to propagate the results in parallel towards the master node.

When running on a computer cluster, gEMpicker parallelises the overall calculation by distributing the work to nodes in the cluster using the MPI (Message Passing Interface) library [35]. At this level of parallelisation, gEMpicker assumes that each node in the cluster has the same hardware configuration so that if the work is divided equally, the main process on each node will finish at approximately the same time. gEMpicker distributes threads to nodes on either a per-micrograph or per-template basis according to the hardware configuration and the actual number of micrographs and templates to be compared. In the per-micrograph scheme, the correlation and index maps for each micrograph are computed by a single node for all templates. Conversely, in per-template mode, all nodes will collaboratively compare all templates with each micrograph. Assuming many templates need to be processed, this mode should be more efficient when the number of CPU cores exceeds the number of micrographs to be processed. However, in order to achieve this gain, the thread-level correlation and index maps need to be collected and combined efficiently. Thus, cluster calculations require an additional reduction step to combine the correlation and index maps from all processes in order to obtain the global NCC map and global IND map using Equations (1) and (2). Figure 1 illustrates the hierarchical parallel structure of gEMpicker running in a multi-node cluster.

We have implemented both direct and tree-based reduction algorithms in gEMpicker. The direct reduction algorithm uses the MPI_Send and MPI_Recv functions to send and receive data between the node and master processes. For a cluster of 2^*n*^ nodes, this approach requires 2^*n*^-1 data transfers and 2^*n*^-1 reduce operations. The tree-structured reduction uses the MPI_Reduce function to propagate results towards the master process at the root of the tree and requires only *n* data transfers and *n* reduce operations in a cluster of 2^*n*^ nodes. Such a tree-based approach is theoretically optimal, since the total elapsed time should scale only logarithmically in the number of cluster nodes. It is worth noting that the cluster reduction step is performed only when all node-level processes have finished their correlation tasks. This means that the reduction calculation itself may be accelerated using multi-threading on each node’s main process. Because this step mainly involves element-wise processing of large arrays it is easily parallelised using a few fine-grained OpenMP [36] compiler directives.

### The computational platforms

In this study, computational experiments were carried out using four different computational platforms, representing four possible hardware configurations. Dirac is a high performance workstation equipped with a contemporary GPU, Mbiserv is a modern GPU server (4 GPUs), Adonis is a small GPU cluster (8 CPU nodes, 16 GPUs), and Griffon is a medium-sized CPU cluster (64 CPU nodes). Dirac, Adonis, and Griffon all have pairs of quad-core CPUs, whereas Mbiserv has two hex-core CPUs. We included Mbiserv in our experiments because it was the only machine available to us which is directly connected to four GPUs. Further details of these machines are given in Table 1. Although gEMpicker uses essentially the same code on all platforms, some of the compiler directives will necessarily differ when compiling it for a CPU or GPU cluster. For academic use, binary versions of gEMpicker are available from the authors on request.

**Table 1:** The characteristics of the four computer platforms used in the current study

**Machine** **name**	**CPU** **cores**	**CPU** **type**	**Memory** **(node)**	**GPUs** **(total)**	**GPU** **type**	**Infiniband** **connection**
Dirac	8	i7-965 (3.2GHz)	12Gb	1	C2075 (575MHz, 448 cores)	–
Mbiserv	12	X5690 (3.5GHz)	64Gb	4	C2075 (575MHz, 448 cores)	–
Adonis	8×8	E5520 (2.3GHz)	24Gb	16	C1060 (602MHz, 240 cores)	40GB/s
Griffon	64×8	L5420 (2.5GHz)	16Gb	0	–	20GB/s

## Results and discussion

### FFT-based NCC performance comparison

**Figure 2:**
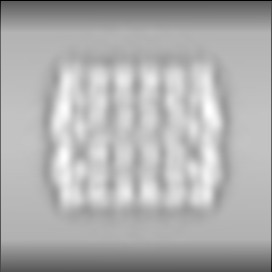
**Comparison of the relative speed of 2D FFT NCC single-precision (a) and double-precision (b) calculations at different micrograph sizes *N×N *(pixels) using the MKL, FFTW libraries on one CPU core of our Dirac workstation (3.2GHz i7-965) and the CUFFT library on one C2075 GPU (448 cores).** The size of template images are chosen as *N*/8×*N*/8. All timings are normalised to the FFTW (one unit).

Figure 2 compares the relative computational speed of 2D NCC calculations using FFTW, MKL, and CUFFT for a range of micrograph sizes using both single and double precision calculations. This figure shows that while MKL is always somewhat faster than FFTW, the speed-up obtained by performing the calculation on a GPU is quite dramatic, especially for large micrographs. For example, for a micrograph of size 4096×4096 and a template image of size 160×160, using CUFFT gives ∼47× speed-up for single precision data and ∼30× speed-up for double precision data. Thanks to recent advances in imaging technology, it is currently common to have digital micrographs of size 2048×2048 or 4096×4096, and the coming generations of EM imaging devices promise to produce even larger sizes. This suggests that the use of GPUs for NCC-based particle picking could be even more advantageous in the near future when even larger micrographs become available. Because it seems that single precision FFT calculations are sufficiently accurate for NCC-based particle picking (see Section Case study: keyhole limpet hemocyanin), all subsequent results will be reported only for single precision calculations.

**Figure 3:**
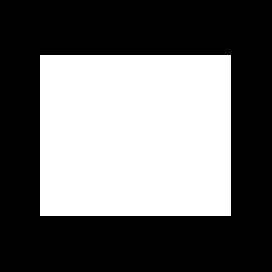
**The speed-up obtained by using multi-threading to calculate 14,630 2D single-precision NCCs of size *4096×4096 *on (a) a dual quad-core workstation (Dirac), and (b) a four-GPU server (Mbiserv).** See Table 1 for hardware details.

To evaluate the multi-threading performance in gEMpicker, the global single precision correlation map between 14,630 template images of size 160×160 and a micrograph of size 4096×4096 was calculated on our Dirac (8-core workstation) and Mbiserv (four-GPU server) machines (see Table 1 for details). The total number of forward and inverse FFTs performed in this case is 73152. The total computation time was ∼7,415s on Dirac and just ∼160s on Mbiserv. This corresponds to an average rate of 0.25 templates/CPU-core/s and 22.9 templates/GPU/s, respectively. Figure 3a shows the relative speed-up for these calculations when using different numbers of CPU cores. This figure shows that the speed-up is almost linear for the first four CPU cores (speed-up ∼3.5×), but that using further cores gives an increasingly smaller gain, and using all 8 cores of a dual quad-core machine gives a speed-up of only about 5.5 ×. This effect is presumably due to the operating system overhead of scheduling multiple threads and their independent memory access patterns on a pair of fully loaded quad-core CPUs. On the other hand, Figure 3b shows that when using up to four GPUs, the speed-up increases linearly with the number of GPUs with apparently no loss of performance as the number of GPUs increases.

### Multi-node cluster performance

**Figure 4:**
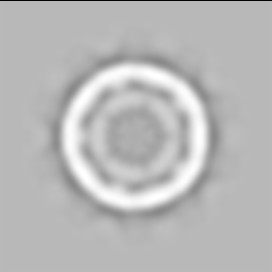
**The speed-up obtained when using (a) a 64-node CPU cluster and (b) an 8-node GPU cluster to calculate 14,630 2D single-precision NCCs of size *4096×4096.*** Here, the number of threads in each process is equal to the number of CPU cores per node in **(a)**, and the number of GPUs per node in **(b)**.

Figure 4 shows the speed-up factor obtained when performing the above calculation (i.e. calculating the 2D single precision correlation maps between a micrograph of size 4096×4096 and 14,630 template images of size 160×160) on the Griffon and Adonis computer clusters using the per-template mode. In this experiment, only the process correlation maps, NCC^*p*^, not the cluster’s global correlation map NCC, were calculated because the latter involves the reduction step, which is considered separately below. Here, the number of consumer threads in each process is equal to the number of CPU cores per node (Figure 4a) or the number of GPUs per node (Figure 4b). Figure 4 shows that the gain increases almost linearly with the number of nodes when clusters have a relatively small size, such as in Adonis. The sub-theoretical gain in Griffon may be due to the use of a network file system to store all template images in a single storage device. Since each node has a producer thread to read template images for its consumer threads, this could lead to contention on the disc device, as discussed above. Nevertheless, the results in Figure 4b also show that the performance of gEMpicker scales linearly with the number of GPUs and the number of nodes in a GPU cluster.

**Figure 5:**
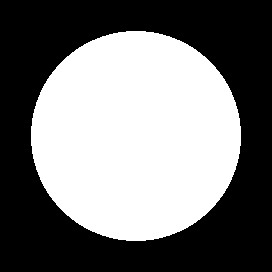
**The reduction time on (a) Griffon (64-node cluster, 20Gb/s InfiniBand), and (b) Adonis (8-node GPU cluster, 40Gb/s InfiniBand) using different inter-process communication strategies (i.e. the MPI_Reduce or MPI_Send and MPI_Recv functions), and different methods for calculating the reduce operation on each node (i.e. with or without using OpenMP parallelisation).** Note that the time axis in **(b)** is 10 × smaller than in **(a)**.

The performance of gEMpicker’s reduction algorithms in the above calculations on the Griffon and Adonis clusters is shown in Figure 5. It can be seen that the total reduction time increases linearly with the number of nodes in direct reduction (MPI_Send/MPI_Recv) and logarithmically with the number of nodes in the tree-based reduction (MPI_Reduce). These observations agree with the expected theoretical performance of these algorithms. Additionally, it can also be seen that using OpenMP to parallelise the reduction inside each process gives a further speed improvement. In particular, this improvement is significantly greater for Adonis (Figure 5b) than Griffon (Figure 5a). This difference can be explained by the higher interconnect speed in the Adonis cluster, which reduces data transfer times and thus exposes the benefit of using OpenMP to accelerate the reduction calculation on each node.

### Case study: keyhole limpet hemocyanin

This section demonstrates the practical utility of gEMpicker using the publicly available keyhole limpet hemocyanin (KLH) dataset^a^. This annotated dataset was used previously to assess the performance of several automatic particle picking algorithms in a particle picking “bake-off” experiment [4]. This relatively small dataset consists of 82 defocus pairs of high-magnification images of size 2048×2048 of KLH particles, the locations of 1042 side-view particles picked manually by a human expert (Mouche’s picks), and a preliminary 3D reconstruction. Each defocus pair contains an image acquired at near-to-focus conditions and an image acquired at far-from-focus conditions.

**Figure 6:**
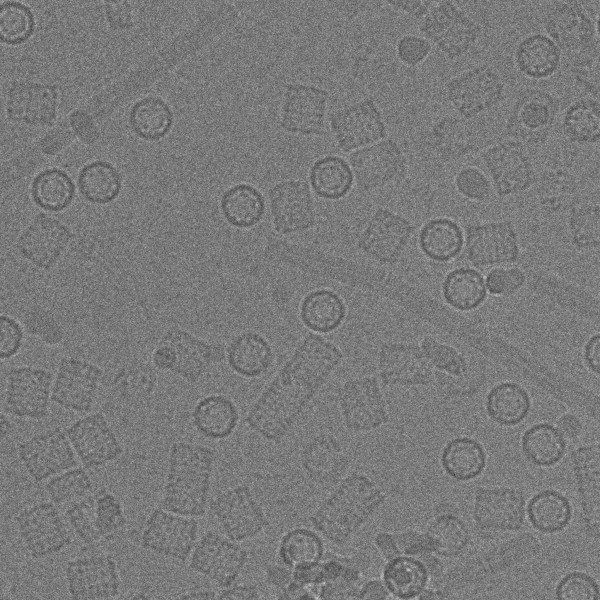
**The templates and masks used in the experiment. (a)** and **(c)**: the side-view and top-view templates obtained by projecting a preliminary 3D density map. **(b)** and **(d)**: the corresponding masks for the two templates used during the NCC calculation.

KLH is a homo-oligomeric didecamer with D5 point group symmetry [37] and exists in two isoforms, KLH1 and KLH2. KLH1 has a short cylindrical shape whereas KLH2, which is usually an aggregate of KLH1, has a longer shape. Since the KLH1 particles usually appear as rectangular side-views with a five-fold axis in the image plane, and as circular top-views with the five-fold axis perpendicular to the image plane, most of the particles in the micrographs may be associated with projections of KLH that correspond to these two views. Thus, the set of templates used in our experiment is generated by projecting the provided preliminary 3D reconstruction in two orientations to produce one side-view and one top-view template (shown in Figures 6b and 6d) using EMAN2 [38] and then rotating the two projections through 360° in 4° intervals to produce a total of 180 images in the template set. For the masks, one rectangular and one circular mask that match the two initial projections (shown in Figures 6b and 6d) were created manually. These masks were then rotated through 360° in steps of 4° to make the corresponding masks for the rotated templates. It should be noted that even though we could benefit the KLH’s symmetry property to effectively reduce the number of templates and masks to 46 without much performance loss, we keep using 180 templates and masks to facilitate comparison with the existing benchmark [8].

**Figure 7:**
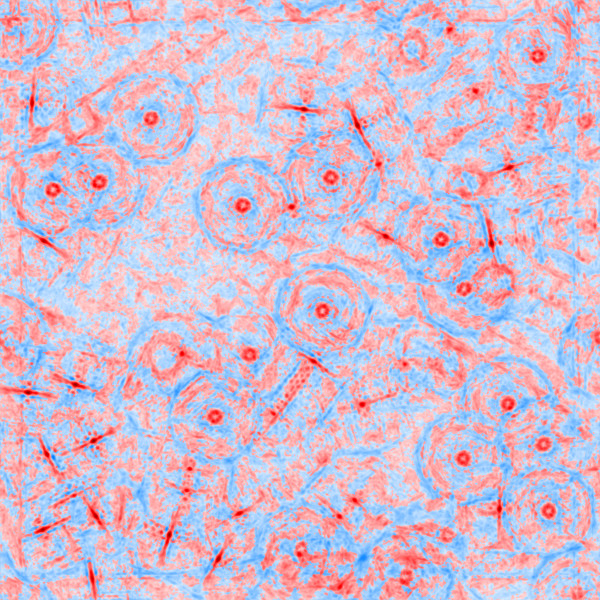
**An example far-from-focus micrograph from the KLH dataset (a), and the calculated global correlation map showing the picked side-view and top-view templates, (b) and (c), which are shown separately for clarity.** The higher peaks (negative values in this case) in the correlation maps correspond to the location of particles. Note that while the side-view templates form rings of relatively high correlation around top-view particles in **(b)**, higher correlation at the centres of these rings are obtained when correlating with top-view templates **(c)**.

**Figure 8:**
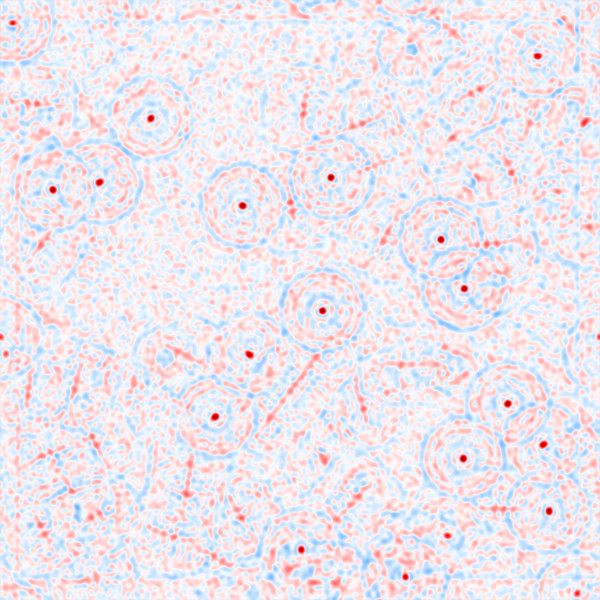
**Receiver-operator-characteristic plot comparison of gEMpicker’s picking performance on the KLH dataset.** Here, the top-scoring 2084 picks from gEMpicker are classified as true or false positives using the 1042 side-view particles manually picked by Mouche (see main text for further details).

In order to suppress intensity variations due to noise, the micrographs of the KLH dataset were convoluted with a 4×4 averaging filter. Each filtered micrograph was then correlated with each of the 180 templates using the algorithm in gEMpicker. The final correlation map for each micrograph contains the global maximum correlation scores for the 180 templates. Finally, gEMpicker extracts the peaks from the resulting 82 global maps using a procedure similar to that of FindEM [8] to identify the template that appears at each peak location. As an example, Figure 7 shows two global correlation maps of a far-from-focus micrograph from the KLH dataset using side-view and top-view templates separately. In these maps, gEMpicker calls a picked particle whenever a local minima of such a map (here, negative values) falls below a certain threshold (-0.3 in this case, similar to the value used in [8]). It is worth noting that a receiver-operator-characteristic (ROC) analysis (see Figure 8) of the results shows that for the KLH dataset using a template rotation step size of 9° gives almost the same level of picking performance as 4°. Thus, correspondingly faster picking speeds may actually be achieved in practice.

We then used gEMpicker to pick particles from the 82 far-from-focus micrographs in the KLH dataset. This gave 1249 side-view particles, which contain 979 (i.e. ∼94%) of Mouche’s 1042 manually picked particles. In comparison, FindEM, which uses Roseman’s NCC algorithm, picked 1282 side-view particles containing 1011 (i.e. ∼97%) of the manually picked particles. Thus, gEMpicker picked approximately 3% fewer particles than FindEM from 3% fewer attempts. The small difference in the results between gEMpicker and FindEM is due to the different templates and masks used here and the slightly different parameter settings in the final peak extraction procedure. As noted by [4], different human experts can pick different sets of particles, and so it is rather difficult to define a “gold standard” for particle picking. Therefore, although FindEM gave amongst the best results in the bake-off comparison, we would not wish to claim that gEMpicker is superior to FindEM. In addition, since the dataset does not provide the coordinates of manually picked top-view particles, we cannot apply a similar performance comparison for the top-view picking results of gEMpicker. To obtain an independent validation of our results, we uploaded the picks obtained by gEMpicker to the 3D Electron Microscopy Benchmark (http://i2pc.cnb.csic.es/3dembenchmark/) for 50 KLH micrographs. This generated the following statistics: Precision: 78.8%; Recall: 93.6%; False Discovery Rate: 21.2%; F-measure: 85.6%; Average distance from manual pick: 4.7 pixels.

Regarding timing, the total time to compute the correlation maps for the 82 micrographs in this dataset was 5,972s when using one CPU core on Dirac compared to 223s using one C2075 GPU. This corresponds to a GPU/CPU speed-up factor of ∼27. However, in this case it is probably fairer to compare one GPU with one quad-core CPU, which reduces the speed-up factor to ∼9. Effectively, the 82 micrographs in this small dataset may be processed in less than 4 minutes using a single GPU or in just over 28 minutes using all 8 cores of a modern workstation. A higher speed-up is expected using a greater number of templates. In contrast, the FindEM program requires 9,430s to compute the 82 correlation maps using one CPU core on Dirac. Thus, the speed-up obtained by using one C2075 GPU in gEMpicker when compared to FindEM is ∼42×.

Assuming the almost linear speed-up demonstrated by our cluster calculations (Figure 5), we estimated that the entire KLH picking exercise could be completed in about 1 minute on our 4-GPU Mbiserv machine. However, the actual observed time is almost 3 minutes. This is because for micrographs of size 2048×2048, the time required to process four templates in four GPUs is less than the time required to read four templates from the storage device. Hence the consumer threads often have to wait for data to become available. In addition, using multi-threading leads to the additional overhead of combining results at the final step. Similar phenomena are also observed on the Adonis and Griffon clusters. Thus, by exploiting GPUs for the particle picking problem, the rate-limiting factor is no longer raw computing power but the bandwidth of the hard disk drives.

## Conclusions

We have presented gEMpicker, a highly parallel multi-threaded cryo-EM particle picking tool which implements Roseman’s NCC matching algorithm on multi-CPU and multi-GPU computer systems. Our results on picking particles in the KLH dataset indicate that gEMpicker performs at least as well as Roseman’s FindEM algorithm. Our computational experiments show that gEMpicker’s automatic particle picking calculation is approximately 30–40 times faster on a contemporary GPU than on a single CPU core. Compared to a quad-core CPU running four gEMpicker threads in parallel, the speed-up from using one contemporary GPU is a factor of ∼9×. We have shown that increasing the number of GPUs speeds up the calculation linearly with almost no additional overhead. We have also demonstrated how the picking task may be distributed over multiple nodes in a computer cluster. On a cluster with a fast Infiniband connection, our tree-based reduction algorithm for combining node-level picks almost eliminates the overhead of distributing the calculation over multiple nodes, and allows the overall calculation speed to increase almost linearly with the available hardware. Thus, the very high picking throughput that is now possible with gEMpicker will help experimentalists to achieve higher resolution 3D reconstructions more rapidly than before.

## Availability and requirements

**Project name:** gEMpicker **Project homepage:**http://gem.loria.fr/gEMpicker.html**Operating system(s):** Linux OS **Programming language:** C++, CUDA **Other requirements:** Boost 1.49 or higher, FFTW 3.3 or higher, CUDA Toolkit 4.2 or higher **License:** Unlimited for academic use **Any restrictions to use by non-academics:** license needed

## Endnote

^a^ Available at http://ami.scripps.edu/redmine/projects/ami/wiki/KLH_dataset_I.

## Competing interests

The authors declare that they have no competing interests.

## Authors’ contributions

TVH - implemented the software, carried out the experiments, made the figures, wrote sections of the manuscript. XC - advised on the parallelisation and reviewed the manuscript. PS - advised on the implementation, proposed the experiments, reviewed and corrected the manuscript. DWR - advised on the implementation and experiments, wrote some sections of the manuscript. All authors read and approved the final manuscript.

## Supplementary Material

Additional file 1 Supplementary materials. Click here for file
