# Supplementary material for: gEMpicker: a highly parallel GPU-accelerated particle picking tool for cryo-electron microscopy
Source: BMC Struct Biol. 2013 Oct 21;13:25. doi: 10.1186/1472-6807-13-25 (PMC3942177; doi:10.1186/1472-6807-13-25)
Supplement: Additional file 1 — Supplementary materials. [file 1472-6807-13-25-S1.pdf]

Supplementary Materials to

gEMpicker: A Highly Parallel GPU-Accelerated Particle Picking Tool for Cryo-Electron Microscopy

Thai V. Hoang[*,1], Xavier Cavin[1], Patrick Schultz[2], and David W. Ritchie[1]

[1]*Inria Nancy, 615 rue du Jardin Botanique, 54600 Villers-lès-Nancy, France*
[2]*IGBMC, 1 rue Laurent Fries, 67404 Illkirch, Strasbourg, France*

June 11, 2013

[*] *Corresponding author*: Inria Nancy, 615 rue du Jardin Botanique, 54600 Villers-lès-Nancy, France
E-mail address: hoangvva@inria.fr, *Telephone*: +33 3 54 95 85 60, *Fax*: +33 3 83 27 83 19.

1 The local normalized cross-correlation

In its basic form, the normalised cross-correlation (NCC) between a micrograph (target image), T, and a template (search image), S, may be calculated as Goshtasby et al. (1984):

$$\text{NCC}(\mathbf{v}) = \frac{1}{N}\sum_{\mathbf{x}} \frac{(S(\mathbf{x}) - \mu_S)}{\sigma_S} \frac{(T^{\mathbf{v}}(\mathbf{x}) - \mu_{T^{\mathbf{v}}})}{\sigma_{T^{\mathbf{v}}}}, \tag{1}$$

where $\sum_{\mathbf{x}}$ denotes summation over all pixel coordinates $\mathbf{x} \equiv (x, y)$, $T^{\mathbf{v}}$ represents a local region of T at a distance $\mathbf{v}$ from the origin having the same size as S, and the quantities $\mu_{T^{\mathbf{v}}}, \mu_S, \sigma_{T^{\mathbf{v}}}$, and σ_S represent the mean and standard deviation of $T^{\mathbf{v}}$ and S pixel intensities, respectively.

Because the pixel intensities $T(\mathbf{x})$ and $S(\mathbf{x})$ in this expression are each individually normalised, it can be seen that the value of NCC($\mathbf{v}$) is independent of any uniform variations in the brightness and contrast of T and S. However, in EM particle picking, the size of the template will often be much smaller than the size of the micrograph. To exploit FFTs in such cases, it is convenient to pad the search image with zeros up to the same size of the target image, and to define a mask image, $M(\mathbf{x})$, of binary values (zero or one) which serves to control the extent of the non-trivial range of the summation:

$$\text{NCC}(\mathbf{v}) = \frac{1}{P}\sum_{\mathbf{x}} \frac{(\tilde{S}(\mathbf{x}) - \mu_{\tilde{S}})}{\sigma_{\tilde{S}}} \frac{(\tilde{T}^{\mathbf{v}}(\mathbf{x}) - \mu_{\tilde{T}^{\mathbf{v}}})}{\sigma_{\tilde{T}^{\mathbf{v}}}}, \tag{2}$$

where P denotes the number of non-zero mask elements, $\tilde{S}(\mathbf{x}) = M(\mathbf{x})S(\mathbf{x})$ and $\tilde{T}^{\mathbf{v}}(\mathbf{x}) = M(\mathbf{x})T^{\mathbf{v}}(\mathbf{x})$ denote the masked versions of $S(\mathbf{x})$ and $T^{\mathbf{v}}(\mathbf{x})$, respectively. By letting $\tilde{S}(\mathbf{x}) = (\tilde{S}(\mathbf{x}) - \mu_{\tilde{S}})/\sigma_{\tilde{S}}$ denote a normalised masked template, and by noting that $\sum_{\mathbf{x}} \tilde{S}(\mathbf{x}) = 0$ by definition, the above expression reduces to

$$\text{NCC}(\mathbf{v}) = \frac{1}{P}\sum_{\mathbf{x}} (\tilde{S}(\mathbf{x})\tilde{T}^{\mathbf{v}}(\mathbf{x}))/\sigma_{\tilde{T}^{\mathbf{v}}}. \tag{3}$$

However, due to the presence of non-uniform noise in EM micrographs and due to the fact that search objects often only occupy a sub-region of S, Roseman (2003) proposed that better correlations may be calculated if the mask array is adapted to match more closely the shape of the search object. For example, a circular instead of a rectangular mask could be used, or indeed the elements of the mask could be set according to the individual pixel intensities of the search object. The utility of using a simple circular mask function has been demonstrated by Rath and Frank (2004).

Clearly, when all of the mask elements are unity, Equation 2 reverts to Equation 1. However, $\sigma_{\tilde{T}^{\mathbf{v}}}$ in Equation 2 must be recalculated for each value of the shift $\mathbf{v}$. Nonetheless, the general expression can still be evaluated using a total of seven FFTs by re-writing it as follows. Firstly, by expanding the denominator we obtain

$$\text{NCC}(\mathbf{v}) = \big(\sum_{\mathbf{x}} \tilde{S}(\mathbf{x})\tilde{T}^{\mathbf{v}}(\mathbf{x})\big)/\big(P\sum_{\mathbf{x}}(\tilde{T}^{\mathbf{v}}(\mathbf{x}) - \mu_{\tilde{T}^{\mathbf{v}}})^2\big)^{1/2}. \tag{4}$$

Then, using the fact that $\sum_{\mathbf{x}} \tilde{T}^{\mathbf{v}}(\mathbf{x}) = P\mu_{\tilde{T}^{\mathbf{v}}}$, the denominator may be expressed entirely as sums:

$$\text{NCC}(\mathbf{v}) = \big(\sum_{\mathbf{x}} \tilde{S}(\mathbf{x})\tilde{T}^{\mathbf{v}}(\mathbf{x})\big)/\big(P\sum_{\mathbf{x}} \tilde{T}^{\mathbf{v}}(\mathbf{x})^2 - (\sum_{\mathbf{x}} \tilde{T}^{\mathbf{v}}(\mathbf{x}))^2\big)^{1/2}. \tag{5}$$

Furthermore, because multiplication by a binary mask does not change the template and because the actions of squaring and translating pixel intensity values obviously commute, this expression may be calculated as

$$\text{NCC}(\mathbf{v}) = A(\mathbf{v})/\big(P \times B(\mathbf{v}) - C(\mathbf{v})^2\big)^{1/2}, \tag{6}$$

where

$$A(\mathbf{v}) = \sum_{\mathbf{x}} \tilde{S}(\mathbf{x}) \times \mathcal{S}(\mathbf{v}) T(\mathbf{x}), \tag{7}$$

$$B(\mathbf{v}) = \sum_{\mathbf{x}} M(\mathbf{x}) \times \mathcal{S}(\mathbf{v}) T(\mathbf{x})^2, \tag{8}$$

$$C(\mathbf{v}) = \sum_{\mathbf{x}} M(\mathbf{x}) \times \mathcal{S}(\mathbf{v}) T(\mathbf{x}), \tag{9}$$

and where $\mathbf{v}$ represents a pixel location and $\mathcal{S}(\mathbf{v})$ represents the operation of translation by $\mathbf{v}$. In other words, the general masked NCC may be calculated for all translational shifts by calculating four forward FFTs (i.e. of $\tilde{S}(\mathbf{x})$, $M(\mathbf{x})$, $T(\mathbf{x})$, and $T(\mathbf{x})^2$), and the three inverse FFTs implied by the products in the above three expressions.

In the EM particle picking problem, it is common that one target image is correlated with multiple search images. In such cases, the forward FFTs of $T(\mathbf{x})$ and $T(\mathbf{x})^2$ need only be calculated once for each set of search images. Hence, when correlating multiple templates, the overall computational cost can be reduced to essentially that of two forward FFTs and three inverse FFTs per template. Furthermore, if a uniform mask is used for all search images, the computational cost essentially reverts to only one forward FFT and one inverse FFT.

References

Goshtasby, A. A., Gage, S. H., Bartholic, J. F., 1984. A two-stage cross-correlation approach to template matching. IEEE Trans. Pattern Anal. Mach. Intell. 6 (3), 374–378.

Rath, B. K., Frank, J., 2004. Fast automatic particle picking from cryo-electron micrographs using a locally normalized cross-correlation function: a case study. Journal of Structural Biology 145 (1–2), 84–90.

Roseman, A. M., 2003. Particle finding in electron micrographs using a fast local correlation algorithm. Ultramicroscopy 94 (3–4), 225–236.
